# New Graduates Encouraged to Submit their Work for Publication

**DOI:** 10.1051/ject/2024012

**Published:** 2024-06-18

**Authors:** Raymond K Wong

**Affiliations:** 1 Associate Professor, The University of Arizona

**Figure 1:**
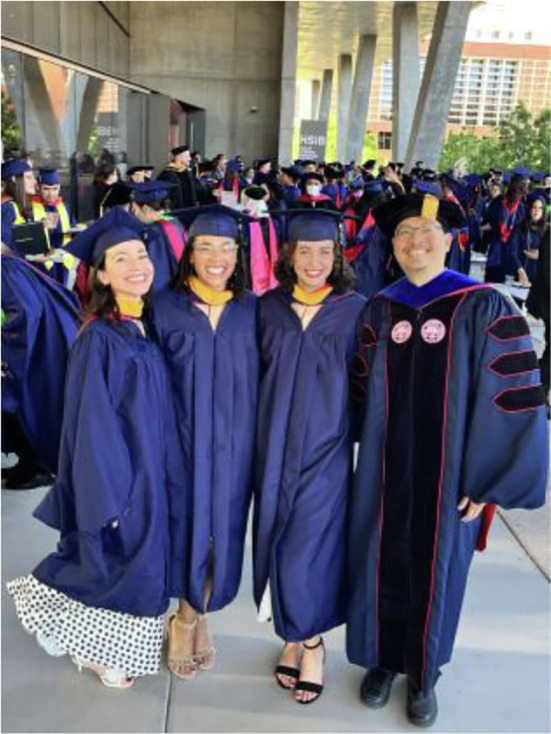
University of Arizona Perfusion Sciences Class of 2024. L-R: Michelle Tigrero, Tylyn Simpson, Madison (Sonny) Lynch and program director, Dr. Raymond K. Wong.

Its graduation season for many US perfusion schools as I am writing this, a time of celebration for the graduates and their families! For me, as a perfusion program director, I am always brimming with pride as I hood my graduates (Figure 1).

They and all other perfusion graduates have overcome many escalating challenges that faculty and preceptors have thrown their way during our training programs in order to shape our trainees to be the best possible new perfusionists they can be. In addition to their didactic and clinical coursework, many motivated and driven students often complete impressive capstone/thesis projects during their time in school. This spring, I was fortunate to attend three major perfusion meetings in the US, the American Academy of Cardiovascular Perfusionists, the American Society of ExtraCorporeal Technology and the Sanibel Symposium. Students presented their work at each of these meetings. Under the guidance of their mentors, some of this work were impressive and definitely worthy of publication! Unfortunately, sometimes this final act of scholarly activity to permanently document one’s work in the literature can fall by the wayside for any number of reasons. These include burnout from school, excitement to start their new careers, challenges of a new steep learning curve as independent clinicians, workload at their new places of employment, focus on national board exam preparations, etc. I get it!

However, graduating seniors should know that having worked so hard to generate data or scour the literature for new insights, adding your work to the perfusion knowledge base can have unexpected, lasting impacts. At a couple of the conferences I attended this year, I presented updates on how JECT has been faring and one of my slides contained two examples of review articles written by former perfusion graduates (Table 1).

**Table 1:** Top 10 most downloaded JECT articles archived on PubMed Central as of March 2024. Source: PubMed Central.

N°	Title	Authors	Year published	Full text requests	PDF requests	Total requests
1	General Anesthesia in Cardiac Surgery: A Review of Drugs and Practices	Cory M. Alwardt, Daniel Redford, Douglas F. Larson	2005	9316	348	9664
2	History and Use of del Nido Cardioplegia Solution at Boston Childrens hospital	Gregory S. Matte, Pedro J. del Nido	2012	8499	888	9387
3	Use of del Nido Cardioplegia for Adult Surgery at the Cleveland Clinic: Perfusion Implications	Kuna Kim, Clifford Ball, Patrick Grady, Stephanie Mick	2014	8557	780	9337
4	Troubleshooting Adult ECMO	David Sidebotham	2011	7061	622	7683
5	Clinical Microsystems: A Critical Framework for Crossing the Quality Chasm	Donald S. Likosky	2014	6629	507	7136
6	Hyperlactatemia and Cardiac Surgery	Jonathon Minton, David S. Sidebotham	2017	5517	664	6181
7	Platelet Mapping by Thromboelastography and Whole Blood Aggregometry in Adult Patients Supported by Mechanical Circulatory Support Device on Aspirin Therapy	Oksana Volod, Francisco A. Arabia, Lee D. Lam, Alice Runge, Caleb Cheng, Lawrence S.C. Czer	2020	5739	232	5971
8	Psychological Depression and Cardiac Surgery: A Comprehensive Review	Phillip J. Tully	2012	4658	132	4790
9	Hyperglycemia as an Effect of Cardiopulmonary Bypass: Intraoperative Glucose Management	Samira Najmaii, Daniel Redford, Douglas F. Larson	2006	4634	119	4753
10	STS/SCA/AmSECT/SABM Update to the Clinical Practice Guidelines on Patient Blood Management	Pierre Tibi, R. Scott McClure, Jiapeng Huang, Robert A. Baker, David Fitzgerald, C. David Mazer, Marc Stone, Danny Chu, Alfred H. Stammers, Tim Dickinson, Linda Shore-Lesserson, Victor Ferraris, Scott Firestone, Kalie Kissoon, Susan Moffatt-Bruce	2021	2939	1058	3997

Cory Alwardt wrote a review article on anesthesia drugs and practices in cardiac surgery in 2005 [1], and Samira Najmaii wrote one on intraoperative management of glucose to treat the hyperglycemic effects of cardiopulmonary bypass in 2006 [2]. These University of Arizona graduates mentored by my predecessor, Dr Doug Larson, could not have realized that almost 20 years later, their work would be the number 1 and 9th most downloaded JECT papers amongst all JECT articles archived at PubMed Central (PMC)! Other most sought after JECT articles from PMC, not surprisingly, are two articles on del Nido cardioplegia, one of which was by Dr del Nido himself with his chief perfusionist, Greg Matte; [3, 4] and two other articles by our Kiwi anesthesiologist and intensivist colleague, Dr Sidebotham, on troubleshooting ECMO and Hyperlactatemia [5, 6]. Also just breaking into the top 10 despite being more recently published is the blood management clinical practice guideline update authored by some of our leading perfusionist colleagues in collaboration with surgeons and anesthesiologists [7]. In actuality, all ten of these articles are on topics that truly remain pertinent to this day.

Back to student authored manuscripts, if students choose to submit review articles, these do need to be extensive and well cited (e.g. >40 references). Research articles will require meticulous work. However, I do want to encourage students to consider putting in just such exhaustive efforts in your work as they can yield meaningful and lasting contributions to our profession as demonstrated above. In conclusion, if you are reading this as a new graduate with publishable work, do consider reengaging with your mentors and submitting your work as soon as you can. JECT and our peer reviewers are standing by to help you ready your work for publication.
